# Comparative study of SRS end‐to‐end QA processes of a diode array device and an anthropomorphic phantom loaded with GafChromic XD film

**DOI:** 10.1002/acm2.13747

**Published:** 2022-08-10

**Authors:** Seng Boh Lim, LiCheng Kuo, Tianfang Li, Xiang Li, Ase M. Ballangrud, Michael Lovelock, Maria F. Chan

**Affiliations:** ^1^ Department of Medical Physics Memorial Sloan Kettering Cancer Center New York New York USA

**Keywords:** end‐to‐end test, quality assurance, radiochromic film dosimetry, SBRT, SRS

## Abstract

**Purpose:**

End‐to‐end testing (E2E) is a necessary process for assessing the readiness of the stereotactic radiosurgery (SRS) program and annual QA of an SRS system according to the AAPM MPPG 9a. This study investigates the differences between using a new SRS MapCHECK (SRSMC) system and an anthropomorphic phantom film‐based system in a large network with different SRS delivery techniques.

**Methods and materials:**

Three SRS capable Linacs (Varian Medical Systems, Palo Alto, CA) at three different regional sites were chosen to represent a hospital network, a Trilogy with an M120 multi‐leaf collimator (MLC), a TrueBeam with an M120 MLC, and a TrueBeam Stx with an HD120 MLC. An anthropomorphic STEEV phantom (CIRS, Norfolk, VA) and a phantom/diode array: StereoPHAN/SRSMC (Sun Nuclear, Melbourne, FL) were CT scanned at each site. The new STV‐PHANTOM EBT‐XD films (Ashland, Bridgewater, NJ) were used. Six plans with various complexities were measured with both films and SRSMC in the StereoPHAN to establish their dosimetric correlations. Three SRS cranial plans with a total of sixteen fields using dynamic conformal arc and volumetric‐modulated arc therapy, with 1–4 targets, were planned with Eclipse v15.5 treatment planning system (TPS) using a custom SRS beam model for each machine. The dosimetric and localization accuracy were compared. The time of analysis for the two systems by three teams of physicists was also compared to assess the throughput efficiency.

**Results:**

The correlations between films and SRSMC were found to be 0.84 (*p* = 0.03) and 0.16 (*p* = 0.76) for γ (3%, 1 mm) and γ (3%, 2 mm), respectively. With film, the local dose differences (ΔD) relative to the average dose within the 50% isodose line from the three sites were found to be −3.2%–3.7%. The maximum localization errors (E_local_) were found to be within 0.5 ± 0.2 mm. With SRSMC, the ΔD was found to be within 5% of the TPS calculation. E_local_ were found to be within 0.7 to 1.1 ± 0.4 mm for TrueBeam and Trilogy, respectively. Comparing with film, an additional uncertainty of 0.7 mm was found with SRSMC. The delivery and analysis times were found to be 6 and 2 h for film and SRSMC, respectively.

**Conclusions:**

The SRS MapCHECK agrees dosimetrically with the films within measurement uncertainties. However, film dosimetry shows superior sub‐millimeter localization resolving power for the MPPG 9a implementation.

## 1 INTRODUCTION

Stereotactic radiosurgery (SRS) and stereotactic body radiation therapy (SBRT) are high‐risk RT techniques delivering a high dose per fraction to small targets, ranging in size from sub‐centimeter to 70 mm.^1^, ^2^ The accuracy of the dosimetry and localization are therefore even more important than normal fractionated treatments to ensure the fidelity of the delivery. End‐to‐end testing (E2E) is a technique to evaluate the treatment workflow from start to finish.^2^ In the context of an SRS/SBRT workflow, E2E can not only be used to detect the dependencies and verify the data transfer among system components but also can be used to evaluate the overall treatment dosimetric and localization accuracy.^1^, ^2^ To assess the readiness of an SRS/SBRT program, MPPG 9a^2^ recommends E2E to be performed annually.

Conventionally, an E2E is performed with a film embedded in an anthropomorphic phantom.^1^ The phantom is scanned, and a plan is developed and delivered in the clinical setting. The exposed E2E film is scanned at high resolution and analyzed to determine the uncertainties in the dosimetry and localization^3^ of the SRS/SBRT workflow. Even with a high throughput film analysis technique,^4^ a significant amount of effort and time are still needed to complete an E2E for a single machine. In a large hospital network with multiple SRS/SBRT type machines, significant resources are then required to perform regular film‐based E2E for all the treatment machines and can be a barrier to comply with the latest recommendations.^2^ Two‐dimensional detector arrays have been shown to be effective in performing patient‐specific QA in lieu of film.^5^, ^6^, ^7^, ^8^ However, the typical detector spacing of this class of detector is in the range of 7–14 mm^7^ which may not be sufficient for SRS measurements.^9^ Recently, the SRS MapCHECK (SRSMC), a two‐dimensional detector array with a detector spacing of 2.47 mm, has become available as an alternative solution to the film‐based E2E test.^10^ However, the performance comparison with film anthropomorphic phantom in a large network has not been investigated. This study investigates the differences between using the new SRSMC system and an anthropomorphic phantom film‐based system in a large network with different SRS delivery techniques.

## 2 MATERIALS AND METHODS

### 2.1 Phantom description

**FIGURE 1:**
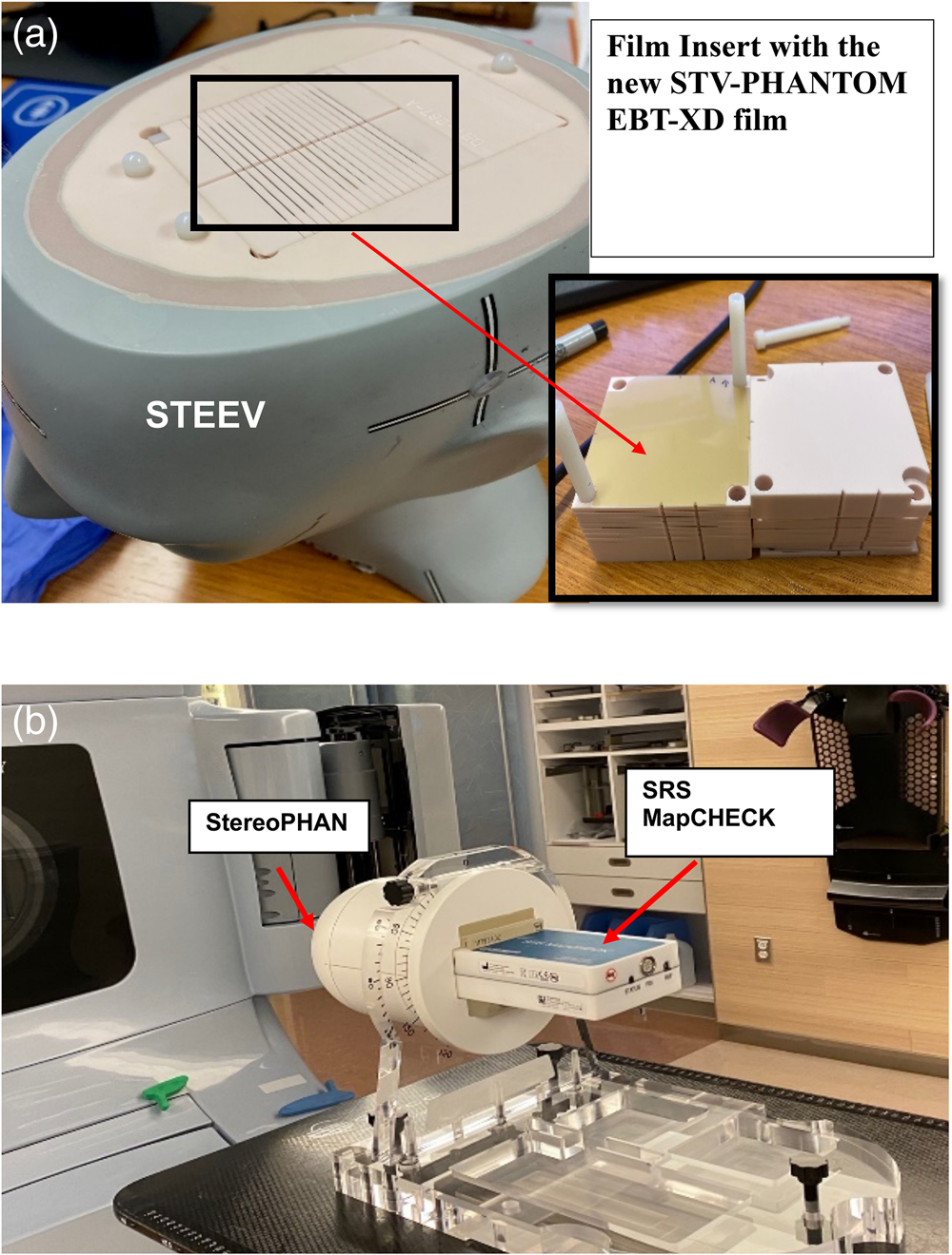
A film‐based anthropomorphic phantom with STV‐PHANTOM EBT‐XD film insert (b) stereotactic radiosurgery (SRS) MapCHECK in StereoPHAN set up on the Protura 6D couch

In this study, the AAPM MPPG 9a^2^ annual SRS dosimetric of 5.0% and localization tolerances of 1.0 mm were used to ensure the fidelity of the whole delivery process. The two phantoms used in this study are the STEEV Phantom from CIRS (Norfolk, VA, USA) and the more recently released SRSMC component for the StereoPHAN from Sun Nuclear Corp (Melbourne, FL, USA). Images of the phantoms are depicted in Figure 1. Both are modular phantoms with various inserts for the user to evaluate different steps of the entire stereotactic process. The phantom's cores and inserts are precision‐milled to ensure high reproducibility of geometry. For a complete description of each phantom as well as all of the inserts available, the reader is referred to the respective manufacturer's websites.

#### 2.1.1 STEEV phantom with film insert

A film‐based anthropomorphic phantom (Figure 1a)‐based E2E test was performed at three clinics in a large hospital network. SRS and SBRT treatments typically have a maximum dose level greater than 10 Gy. To accommodate this, the EBT‐XD film, which has a wider dynamic range^11^ of up to 40 Gy, was used in this study instead of the more standard EBT3 film. The new STV‐PHANTOM EBT‐XD films (Ashland, Bridgewater, NJ), which were made with EBT‐XD films laser cut to fit the STEEV film insert, were used in this study. These high‐precision film edges were used to determine the localization uncertainties as the STEEV film insert does not have any fiducials for registration.

#### 2.1.2 StereoPHAN and SRS MapCHECK

SRSMC‐based E2E tests (see Figure 1b) were performed at three clinics of a large hospital network.

### 2.2 CT and dose calculation

**FIGURE 2:**
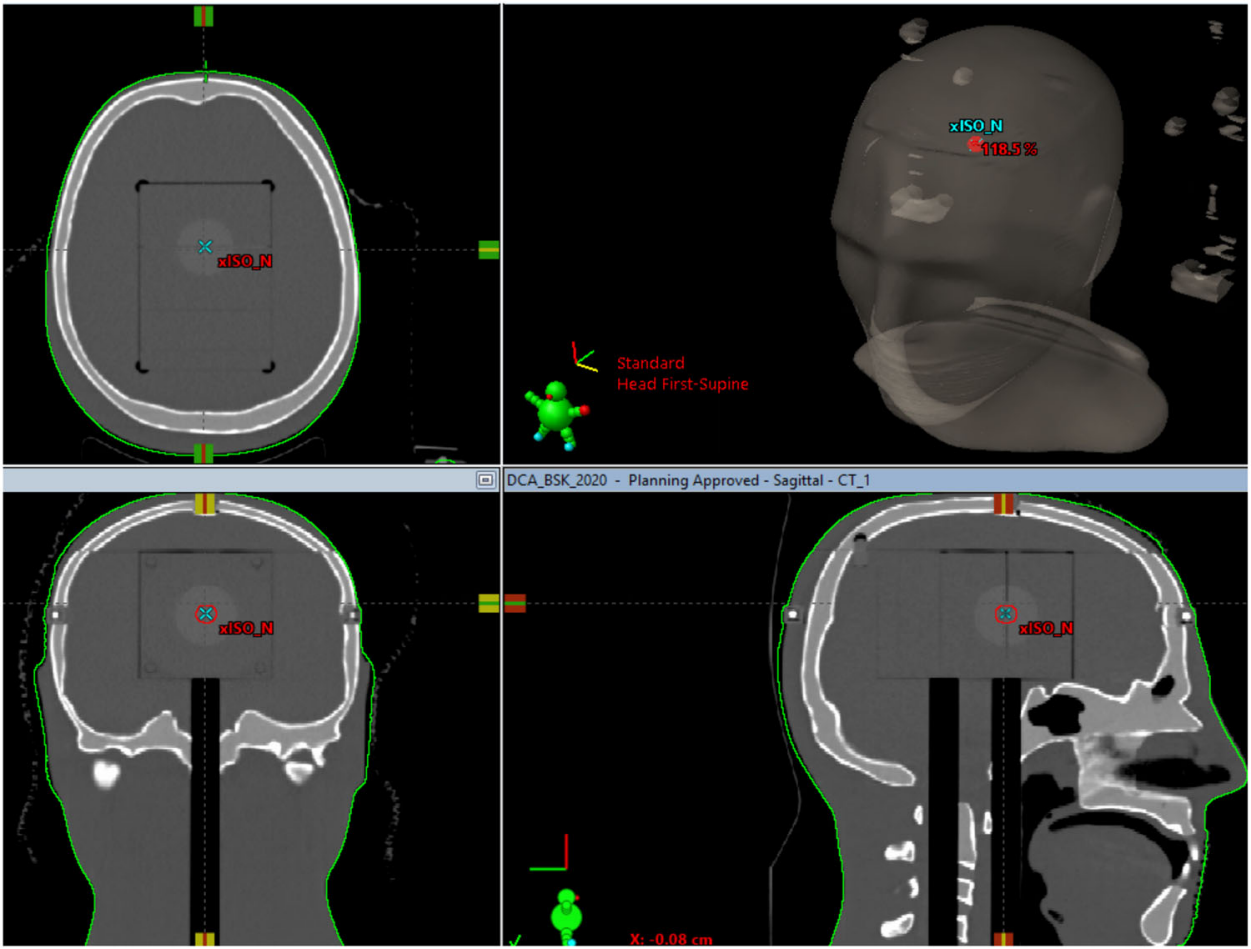
Three orthogonal views of CT imaging of the STEEV phantom

**FIGURE 3:**
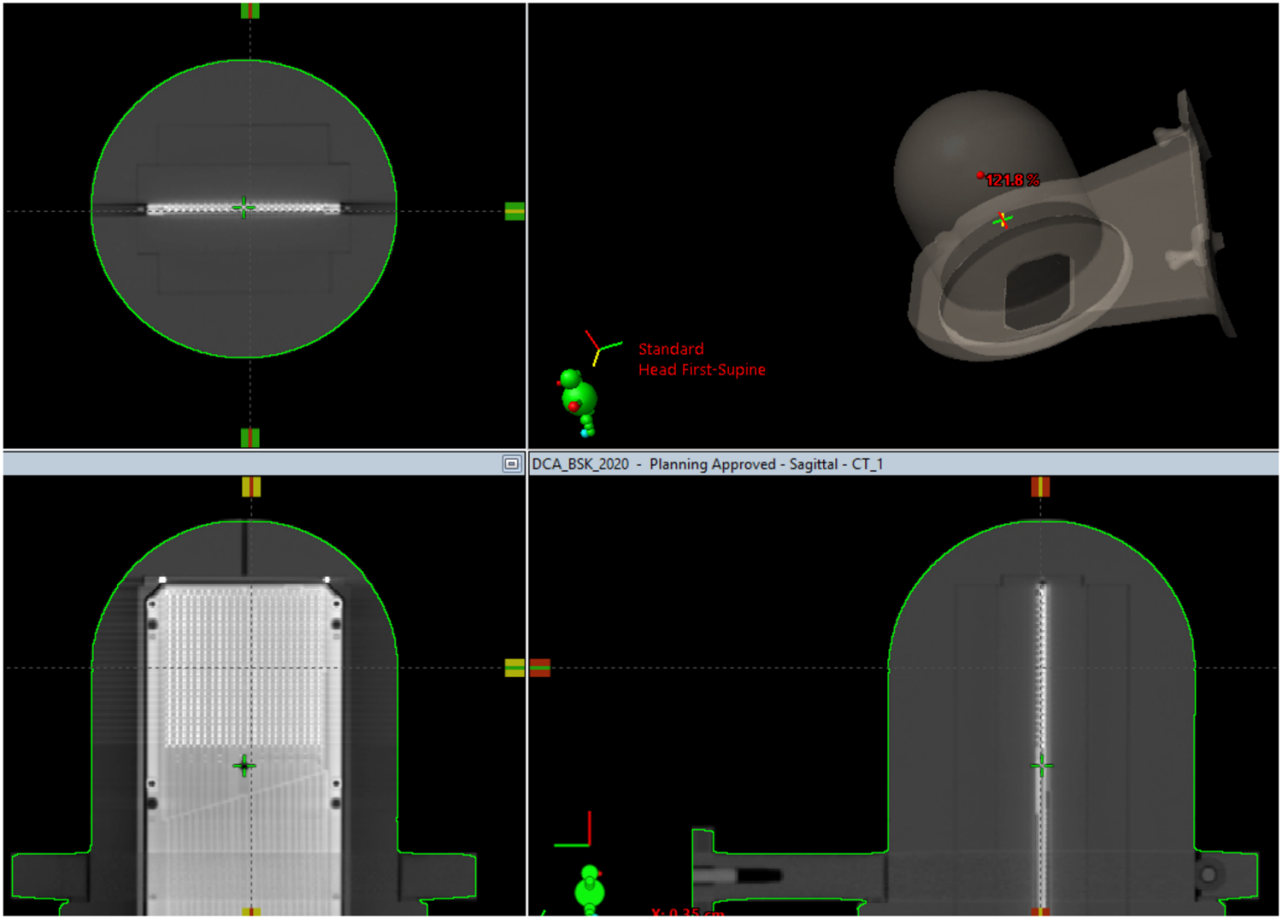
Three orthogonal views of CT imaging of the StereoPHAN/stereotactic radiosurgery (SRS) MapCHECK

Both STEEV with the new STV‐PHANTOM EBT‐XD films embedded and the StereoPHAN/SRSMC phantoms were scanned in 1.0 mm slice resolution and 16‐bit dynamic range with a Phillips Brilliance CT scanner at each site. Figures 2 and 3 show examples of the scanned STEEV and SteroPHAN phantoms, respectively. No hounsfield unit override was used for the STEEV phantom. The density of StereoPHAN was assigned to be 1.2 g/cm^3^, equivalent to acrylic (PMMA), as recommended by the vendor.^12^

### 2.3 Treatment planning and delivery techniques

Three SRS capable Linacs (Varian Medical Systems, Palo Alto, CA) at three different sites were chosen to represent a hospital network: a Trilogy with an M120 multi‐leaf collimator (MLC), a TrueBeam with an M120 MLC, and a TrueBeam Stx with an HD120 MLC. SRS cranial plans using dynamic conformal arc (DCA) and volumetric‐modulated arc therapy, with 1–4 targets, were planned with Eclipse v15.5 treatment planning system (TPS) using a custom SRS beam model for each machine. These plans were generated with the prescription of 23 Gy in a single fraction. The complexity of these plans was assessed using the edge metric^13^ (EM) and the plan irregularity^14^ (PI) indices. EM of each beam is defined as:

(1) $$\mathrm{EM}\;=\sum_{i\;=\;1}^{S}W_{i}\frac{y_{i}}{A_{i}}\;$$

S, W_i_, y_i_, and A_i_ are the total number of segments, aperture weight of each segment, aperture size perpendicular to the MLC motion of each segment, and area of each segment respectively. PI is defined as:

(2) $$\mathrm{PI}\;=\;\frac{\sum_{i\;=\;1}^{B}MU_{i}BI_{i}}{MU_{plan}}$$

B, monitor unit (MU)_i_, BI_i_, and MU_plan_ are the total number of fields in a plan, monitor unit of each field, beam irregularity of each field, and total monitor unit of a plan, respectively.

Both TrueBeam Linacs were equipped with a Varian 6 degree of freedom couch. The Varian Trilogy was equipped with a CIVCO's Protura couch.^15^

#### 2.3.1 DCA for single spherical lesion SRS

**FIGURE 4:**
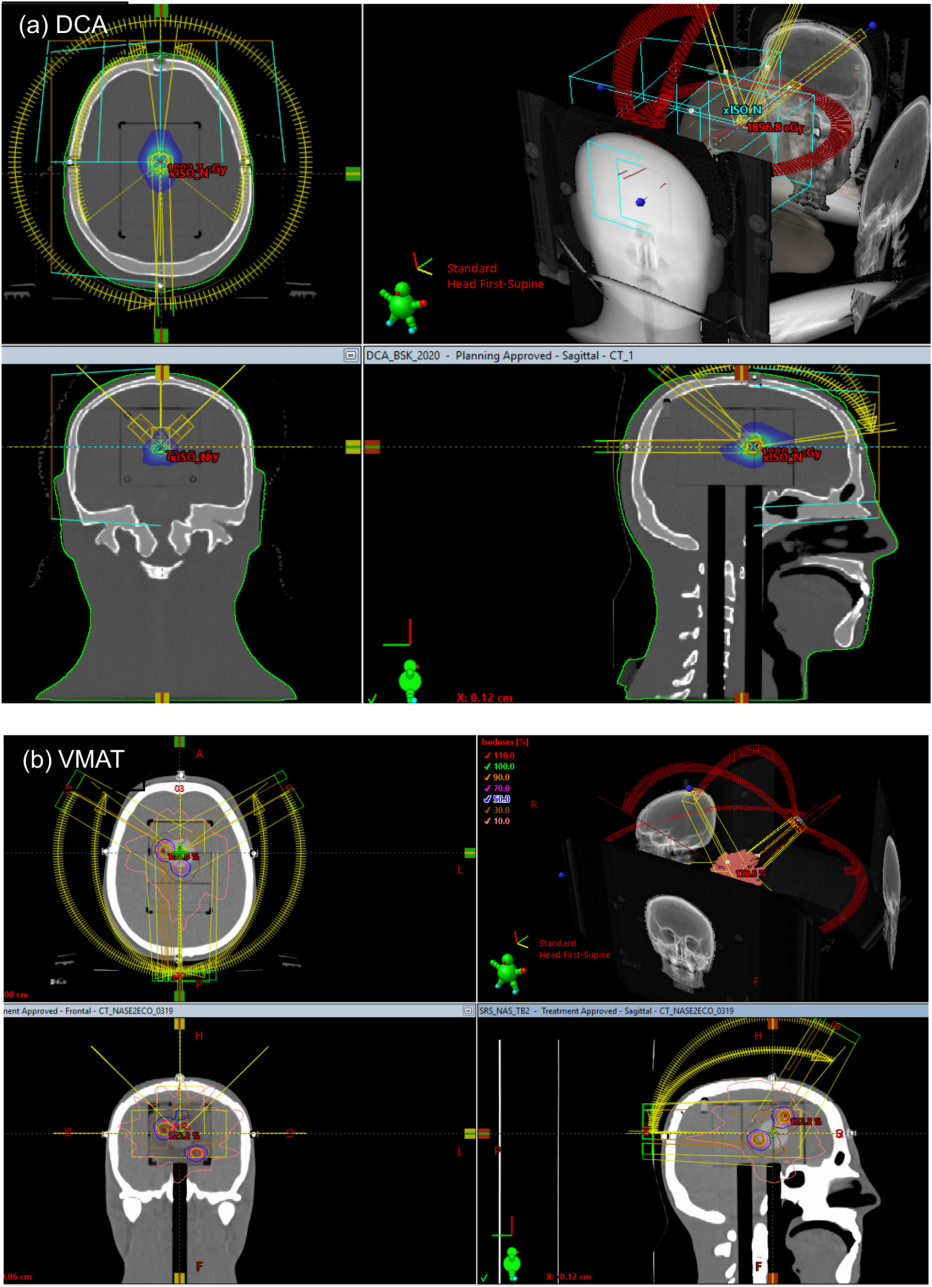
Stereotactic radiosurgery (SRS) planning using (a) dynamic conformal arc (DCA), and (b) VMAT techniques on the STEEV phantom

**FIGURE 5:**
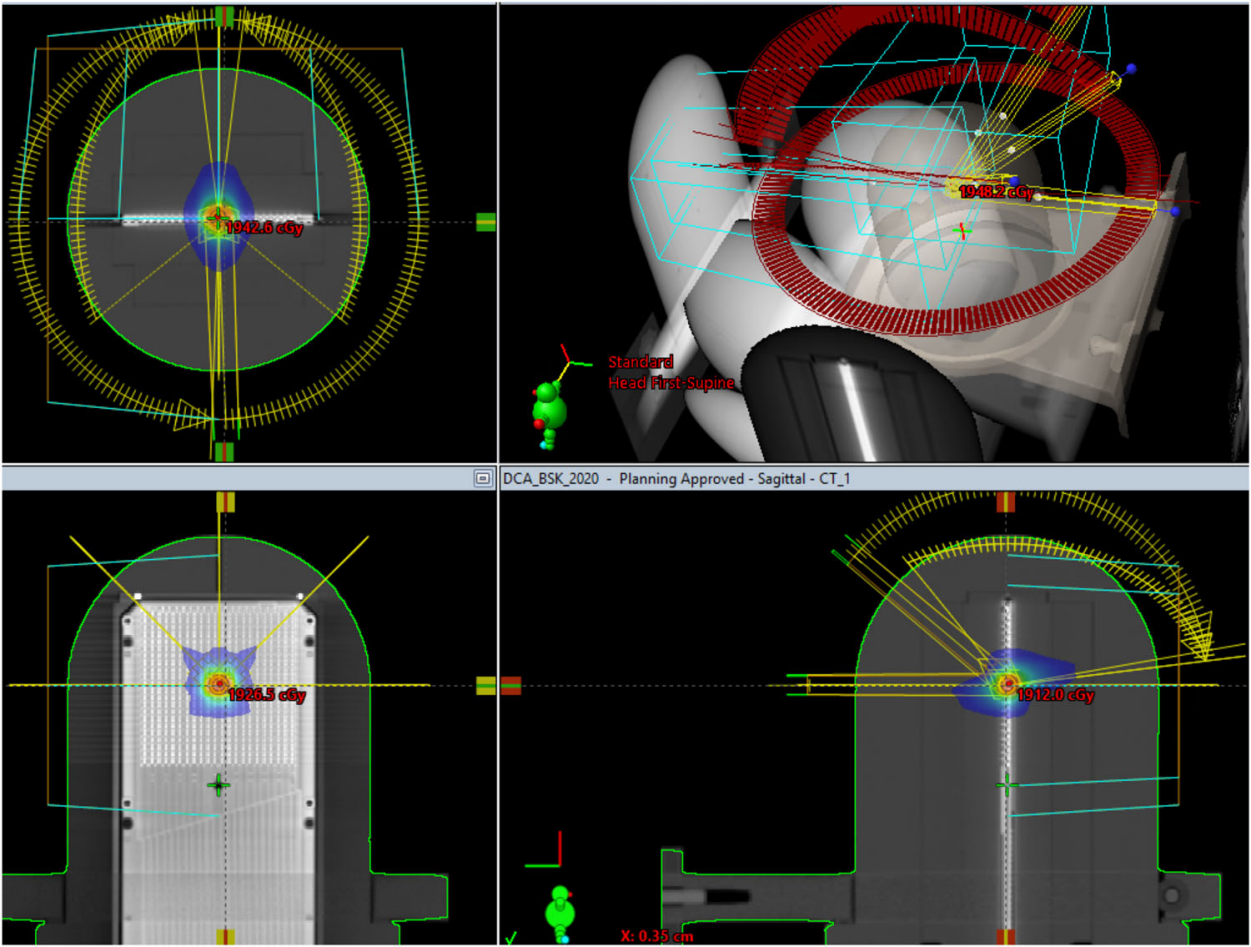
Stereotactic radiosurgery (SRS) planning using dynamic conformal arc (DCA) technique on the StereoPHAN

A single target (∼1 cm size) noncoplanar DCA plan was created using the custom model with (0,0) focal spots in Eclipse v15.5(Varian, Palo Alto, CA, USA). The DCA plan was measured with EBT‐XD film in a STEEV (Figure 4a) and StereoPHAN (Figure 5) at a Varian Trilogy with M120 MLC.

#### 2.3.2 VMAT for multi‐lesion SRS

Multiple targets (1.0–3.0 cm in size) noncoplanar VMAT plans were also created using the custom models in Eclipse v15.5 (Figure 4b). The focal spots for HD120 and M120 MLC machines are (0,0) and (1.75, 0.75) respectively. All VMAT plans were delivered on TrueBeam Linacs. Table 1 summarizes the planning parameters of the plans used in the study.

**TABLE 1:** Planning parameters of the plans used in the study

			Ave. Jaw Size (cm)			Target			Complexity	
Plan	Machine	Fields No.	X	Y	MU	Number	Min. diameter (cm)	Max. diameter (cm)	EM	PI
1	TB HD120	4	4.4	4.8	5362	4	1.1	1.3	0.312	4.728
2	TB M120	4	4.8	5.1	5192	4	1.0	1.3	0.265	5.628
3	CL M120	4	1.3	1.4	2989	1	1.2	1.2	0.168	1.512

Abbreviations: EM, edge metric; PI, plan irregularity.

### 2.4 E2E testing

The dosimetric and localization accuracy were compared. The time of analyzing the two systems by three teams of physicists was also compared to assess the throughput efficiency.

#### 2.4.1 Image guidance reproducibility in a frameless environment with kV‐CBCT

The STEEV phantom with the STV‐PHANTOM EBT‐XD films was set up on the treatment couch, and 2D kV images were taken initially to line up based on the “bony structures,” and then CBCT was taken with the 6D registration with the planning CT images. Similarly, 2D kV and CBCT were taken for the StereoPHAN/SRSMC setup. However, unlike the STEEV phantom, StereoPHAN does not have bony anatomy. The CBCTs were aligned to the CT based on the detector positions inside the SRSMC.

#### 2.4.2 Aperture area metrics

The planning targets in this study ranged from 1.0 to 9.0 cm^3^ in size. These targets were placed within the 50 mm on the same plane from the isocenter.

#### 2.4.3 Leaf and gantry speed metrics

The speeds of the MLC and gantry were limited to 2.5 cm per second and 6 degrees per second in the TPS and the delivery.

#### 2.4.4 Dosimetric and localization analysis

**FIGURE 6:**
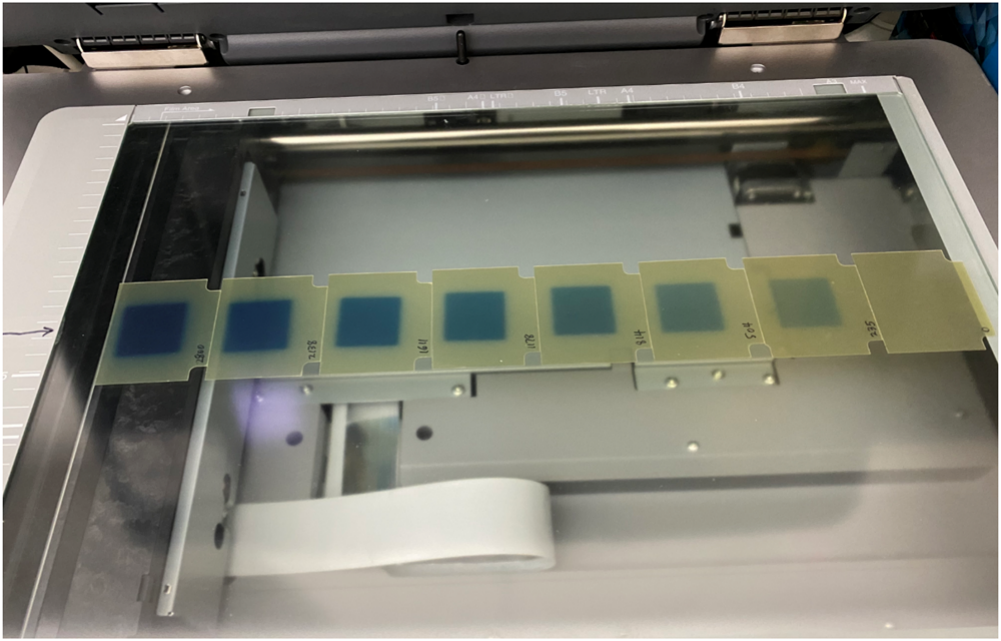
Calibration films scanned on an Epson XL10000 scanner

The dosimetric agreement between the measurements and the TPS calculation (TPSC) was assessed by taking the dose difference (ΔD). The region of interest (ROI) used for the analysis was defined to be the region encompassed by the 50% isodose line of the maximum dose. In the film‐based E2E, the film measurements were registered to the TPS by minimizing the dose difference within the ROI. In addition, the agreement between measurements and TPSC was evaluated using gamma^7^ analysis (γ) with (3%, 2 mm) and (3%, 1 mm) metrics with a 10% threshold. This was performed with an in‐house program. The localization errors (E_local_) were then determined by the shift of the film location relative to its position in the CT scans. All the films were scanned with a one‐scan protocol^4^ in the recommended resolution^16^ of 150 dpi with Epson 10000XL (Epson, Los Alamitos, CA) (see Figure 6). The films were calibrated using the triple‐channel^17^ calibration technique with FilmQA Pro (Ashland, Bridgewater, NJ). The localization errors for SRSMC were determined by the SNC Patient (Sun Nuclear, Melbourne, FL) by locating the best dosimetric fit between the measurements and the TPSC. The dosimetric variations of films and SRSMC were assessed by taking the average and range of the measurements from the three centers. An additional six patient plans, three DCA, two SRS VMAT, and one SBRT spine, were also measured with both films and SRSMC in the StereoPHAN. The gamma results, with 3%/1 and 3%/2 mm, from both detectors were compared using Spearman correlation^18^ to establish the relationship.

**FIGURE 7:**
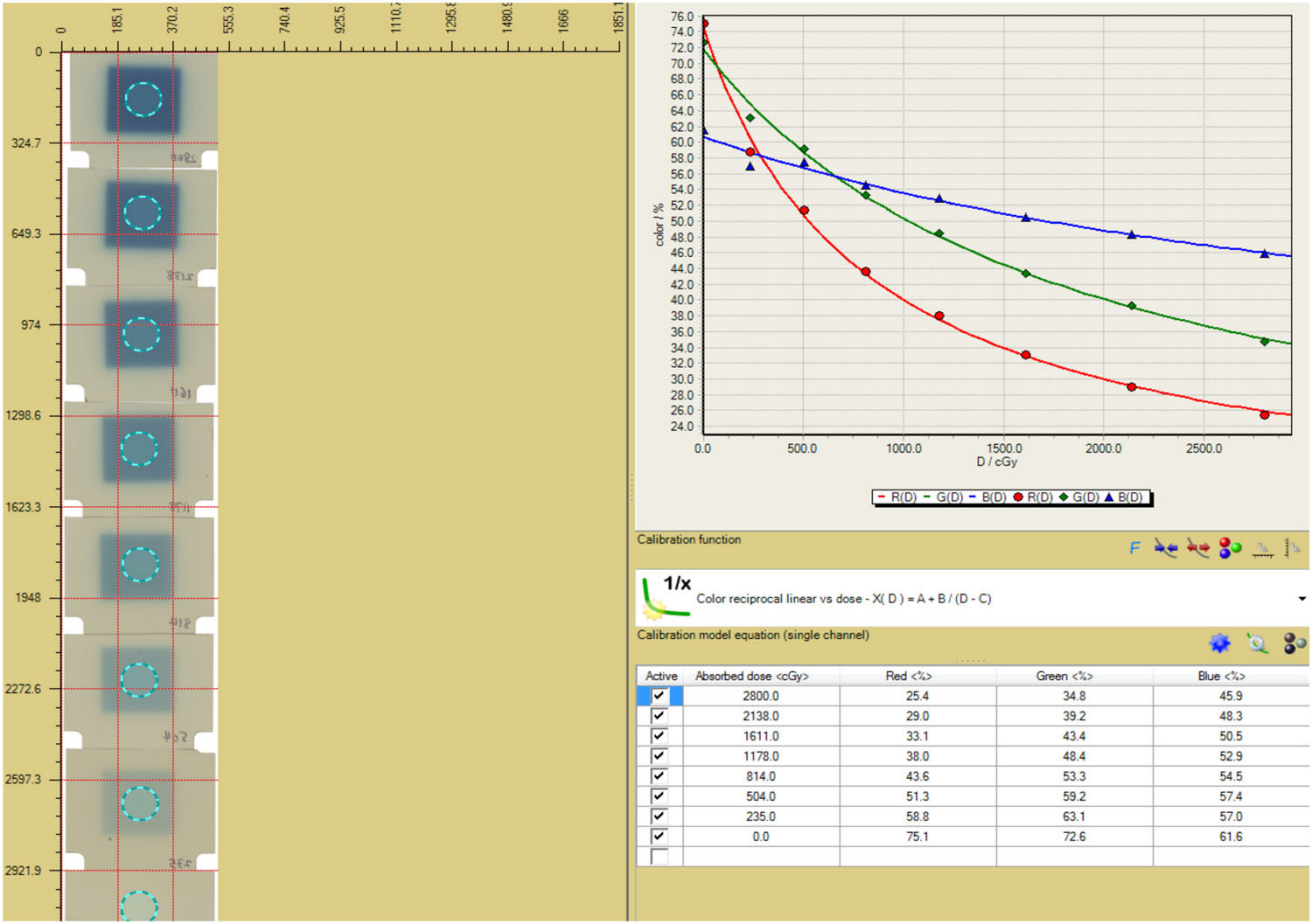
An example of the triple‐color calibration curves using one‐scan protocol

To reduce dosimetric uncertainties at the high dose region, where targets are usually located, a total of eight calibration points, ranging from 0.0 to 28.0 Gy, were used to define the calibration curve. Each calibration film, corresponding to one dose level, was irradiated individually in a water‐equivalent phantom at a depth of 5 cm. The field size of 3 × 3 cm^2^ defined by MLC and jaws, instead of the conventional 10 × 10 cm^2^, was used to expose all the calibration films to minimize the drastic output factor change.^16^ Figure 7 shows the screenshot of the FilmQA Pro 2017 calibration curves for EBT‐XD films with Varian Trilogy Linac (6 MV) up to 28 Gy.

## 3 RESULTS

### 3.1 E2E testing

The correlations between films and SRSMC in the StereoPHAN (Table 2) were found to be 0.84 (*p* = 0.03) and 0.16 (*p* = 0.76) for γ (3%, 1 mm) and γ (3%, 2 mm), respectively. With film, the ΔD from the three sites were found to be in the range from −3.2% to 3.7%. The EM of these E2E ranged from 0.168(DCA) to 0.312(VMAT SRS) (Table 1). The corresponding PI ranged from 1.512 to 4.728. The corresponding results of γ (3%, 2 mm) and γ (3%, 1 mm) were found to be in the range of 98.3%–100.0% and 94.0%‐100.0%, respectively (Table 3A). The scanning resolution of the films was performed at 150 dpi resulting in an estimated uncertainty of around 0.1 mm. The maximum E_local_ values for film were found to be within 0.5 ± 0.2 mm (Table 3B). With SRSMC, the ΔD was found to be within 1.9 % of the TPS calculation (Table 3A). E_local_ values for SRSMC were found to be within 0.7–1.1 mm for TrueBeam and Trilogy, respectively. Comparing with film, an additional average and maximum translational uncertainty of 0.3 and 0.8 mm respectively was found with SRSMC (Table 3B). In terms of rotational agreements in pitch (p), roll (r), and yaw (y) directions, measurements from both techniques were found to be within 0.5° from the corresponding plans and each other. The delivery and analysis times were found to be 6 and 2 h for film and SRSMC, respectively.

**TABLE 2:** Dosimetric error comparisons between film and stereotactic radiosurgery MapCHECK (SRSMC) in the StereoPHAN

		Film		SRSMC	
Machine	Plan	γ (3%, 2 mm)	γ (3%, 1 mm)	γ (3%, 2 mm)	γ (3%, 1 mm)
TB HD120	DCA1	100.0	99.5	100.0	100.0
	DCA2	100.0	100.0	100.0	100.0
	SRS VMAT	100.0	99.6	97.0	96.4
TB M120	DCA1	100.0	100.0	100.0	100.0
	SRS VMAT	99.4	99.1	98.8	97.7
	SBRT Spine	99.0	97.8	98.9	94.5

**TABLE 3A:** Dosimetric error comparisons between anthropomorphic phantom with film and stereotactic radiosurgery MapCHECK (SRSMC)

	Film			SRSMC		
Machine	ΔD (%)	γ (3%, 2 mm)	γ (3%, 1 mm)	ΔD (%)	γ (3%, 2 mm)	γ (3%, 1 mm)
TB HD120	3.7	99.1	95.2	1.5	100.0	99.1
TB M120	−3.1	98.3	94.0	−0.5	100.0	98.3
CL M120	−3.2	100.0	100.0	−1.9	100.0	100.0

**TABLE 3B:** Localization error comparisons between anthropomorphic phantom with film and stereotactic radiosurgery MapCHECK (SRSMC)

	Film E_local_ (x, y, z in mm, p, r, y in degree)						SRSMC E_local_ (x, y, z in mm, p, r, y in degree)					
Machine	x	y	z	p	r	y	x	y	z	p	r	y
TB HD120	0.0	0.0	0.3	0.2	0.0	0.0	0.0	0.7	0.4	0.2	0.5	−0.1
TB M120	0.2	0.5	0.3	0.2	0.0	0.4	0.2	0.0	0.8	0.5	0.0	0.3
CL M120	0.4	0.3	0.4	0.3	0.0	0.2	0.9	1.1	0.9	0.0	0.0	0.2

**FIGURE 8:**
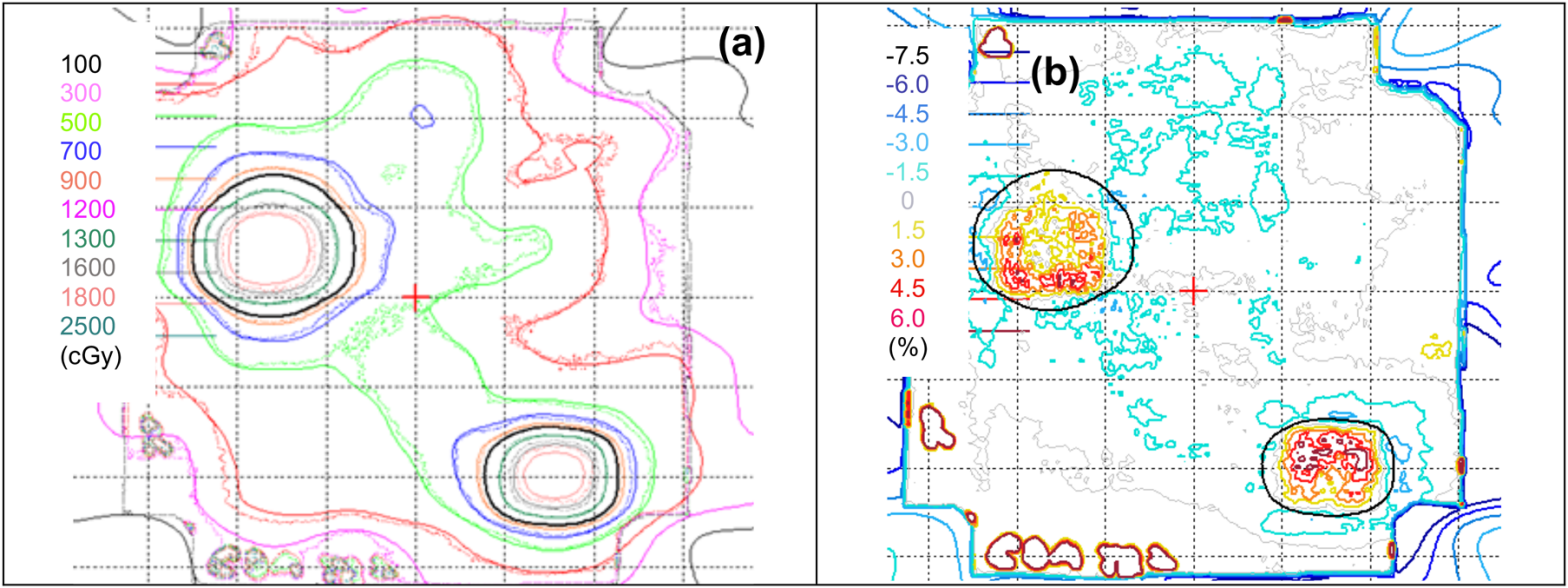
Film‐based anthropomorphic phantom E2E results: (a) dose distribution overlay of film (dotted) and TPS calculation (TPSC) (solid); (b) ΔD of film and TPSC

Figure 8a shows an example of an overlay of the film and the TPSC. Figure 8b shows the corresponding dose difference (ΔD) analysis. Table 1 shows the dosimetric and the localization error, E_local_, of the systems. Overall, the average dosimetric variations of film and SRSMC among centers were found to be −0.3% and −0.2% respectively. The dosimetric ranges of both systems were found to be ±3.2% and ±1.9% for film and SRSMC, respectively. In terms of gamma analysis, film exhibited a larger variation and overall passing score than SRSMC. The E_local_ of the SRSMC showed about 0.7 mm larger deviation than the film results. In terms of time, the delivery and analysis time for film and SRSMC were found to be 6 and 2 h, respectively. The higher time for film‐based E2E was attributed to the extra time required to perform film calibration and the manual analysis needed for both film dosimetry and localization. The shorter turnaround time of SRSMC provides a higher efficiency workflow. For the MPPG 9a application, however, the localization of uncertainty of SRSMC will require further investigation in terms of reproducibility and target size sensitivity.

## 4 DISCUSSION

Significant correlations between films and SRSMC were found with γ (3%, 1 mm) analysis, while no statistically significant correlation was found with γ (3%, 2 mm) in the StereoPHAN study. This is likely a result of the difference in the detector spacing. This should be further investigated but is beyond the scope of this current study. Further, good dosimetric agreements, within 1.9 % dose difference, were found between film and SRSMC with low modulation DCA plans. However, larger dosimetric deviations between the two detectors were also observed with higher complexity plans. Based on the results of this study, SRSMC clearly was able to perform the E2E in an efficient fashion. Similar to another study,^10^ the E2E can be completed three times faster by the SRSMC than films. This is a very appealing advantage for a large hospital network that can benefit from a cost‐effective solution to perform E2E to be in compliance with MPPG 9a. At the recommended resolution^16^ of 150 dpi scanning resolution for SRS dosimetry, the film can effectively provide 0.17‐mm resolution, which is about an order of magnitude higher than the detector spacing of the SRSMC. As a result, film still can provide superior local dose distribution information, such as localized hot and cold spots, when a plan is highly modulated. This can be beneficial to physicists to gain better insights into the limitations of the TPS and the treatment delivery. For example, the slight penumbra mismatch could be easily observed in the film dosimetry but could not easily be appreciated in the SRSMC. This is an important aspect^19^ when clinical physicists are commissioning a TPS or new treatment modality. For example, the small fields (1 × 1 to 3 × 3 cm^2^) output factors of all three machines in this study were first measured with a razor diode (IBA, Louvain‐La‐Neuve, Belgium), and compared with the TPS to less than 0.8% during the preclinical phase. Although the average dose difference was found to be similar between the two detectors, a larger variation of gamma analysis in the film, stemming from the disagreement in the penumbra regions, was observed. Similar mismatches in the penumbra (∼0.6 mm) were observed in the preclinical measurements with razor diode in the phantom. The same information, however, may not be captured in the SRSMC, due to its relatively lower spatial resolution,^20^, ^21^ and resulted in lower gamma analysis sensitivity in high dose gradient in small fields. Similar to a recent study,^10^ the film was found to provide better E2E localization agreement than SRSMC by as much as 0.8 mm. Again, this can likely be caused by the interpolation uncertainty and the larger detector spacing of the device. Moreover, we did not apply the lateral response artifact (LRA) correction^22^ to the SRS E2E film scanning due to the small field sizes used and the nature of less LRA of EBT‐XD film at higher doses (i.e., >10 Gy).^23^ Overall, SRSMC was found to be sufficient for routine QA or subsequent annual SRS E2E testing only after a baseline was established at commissioning with a higher resolution device. Film dosimetry was found to be appropriate for both routine QA and commissioning work because of its high resolution. As this study was based on measurements performed in three centers, more data will be collected to assess the reproducibility and precision of the SRSMC.

## 5 CONCLUSIONS

In this study, we have investigated the differences between using a new SRSMC system and an anthropomorphic phantom film‐based system with different SRS delivery techniques in a large cancer institution network. The SRSMC agrees dosimetrically with the films within measurement uncertainties and provides results rapidly that can be used for annual E2E tests recommended by the AAPM‐RSS MPPG 9a. However, film dosimetry shows superior sub‐millimeter localization resolving power, which should be used for the SRS program end‐to‐end commissioning and cross‐validation to establish QA baselines.

## AUTHOR CONTRIBUTIONS

S.L. conceived of the presented idea. S.L., M.C., L.K., and T.L. performed the experiments. A.B., M.C., L.K., and X.L. evaluated the treatment plans. S.L. performed the data analysis and wrote the manuscript with the support from M.C. and D.M.L. All authors discussed the results and contributed to the final manuscript.

## CONFLICT OF INTEREST

Maria Chan has a research grant from Ashland Inc., the manufacturer of GafChromic film. Other authors have no conflict of interest to disclose.

## Data Availability

Research data are not shared.
